# Using an emergency response infrastructure to help women who experience gender-based violence in Gujarat, India

**DOI:** 10.2471/BLT.15.163741

**Published:** 2016-05-02

**Authors:** Jennifer A Newberry, Swaminatha Mahadevan, Narendrasinh Gohil, Roma Jamshed, Jashvant Prajapati, GV Ramana Rao, Matthew Strehlow

**Affiliations:** a Department of Emergency Medicine, Stanford University School of Medicine, 300 Pasteur Drive, Alway Building M121, Stanford, CA 94305, United States of America (USA).; b GVK Emergency Management Research Institute, Hyderabad, India.

## Abstract

**Problem:**

Many women who experience gender-based violence may never seek any formal help because they do not feel safe or confident that they will receive help if they try.

**Approach:**

A public–private-academic partnership in Gujarat, India, established a toll-free telephone helpline – called 181 Abhayam – for women experiencing gender-based violence. The partnership used existing emergency response service infrastructure to link women to phone counselling, nongovernmental organizations (NGOs) and government programmes.

**Local setting:**

In India, the lifetime prevalence of gender-based violence is 37.2%, but less than 1% of women will ever seek help beyond their family or friends. Before implementation of the helpline, there were no toll-free helplines or centralized coordinating systems for government programmes, NGOs and emergency response services.

**Relevant changes:**

In February 2014, the helpline was launched across Gujarat. In the first 10 months, the helpline assisted 9767 individuals, of which 8654 identified themselves as women. Of all calls, 79% (7694) required an intervention by phone or in person on the day they called and 43% (4190) of calls were by or for women experiencing violence.

**Lessons learnt:**

Despite previous data that showed women experiencing gender-based violence rarely sought help from formal sources, women in Gujarat did use the helpline for concerns across the spectrum of gender-based violence. However, for evaluating the impact of the helpline, the operational definitions of concern categories need to be further clarified. The initial triage system for incoming calls was advantageous for handling high call volumes, but may have contributed to dropped calls.

## Résumé

**Problème:**

Nombre de femmes victimes de violences sexistes ne cherchent aucune aide formelle, car elles ne se sentent pas suffisamment en sécurité ou ne sont pas sûres que leur démarche leur permettra réellement d’obtenir de l’aide.

**Approche:**

Dans le Gujarat, en Inde, un partenariat entre les secteurs public, privé et universitaire a instauré un numéro d'appel gratuit (baptisé 181 Abhayam) pour les femmes victimes de violences sexistes. Ce partenariat s'est appuyé sur une infrastructure existante de réponse d'urgence pour mettre en lien les femmes avec un service d'assistance par téléphone, les organisations non gouvernementales (ONG) et les programmes gouvernementaux.

**Environnement local:**

En Inde, la prévalence sur la vie entière des violences à l'égard des femmes est de 37,2 %. Or, moins de 1 % des femmes cherchent à obtenir de l'aide en dehors du cercle de leur famille ou de leurs amis. Avant l'instauration de ce service téléphonique, il n'existait aucun numéro d'appel gratuit ni aucun système centralisé de coordination pour les programmes gouvernementaux, les ONG et les services de réponse d'urgence.

**Changements significatifs:**

Cette ligne téléphonique a été lancée en février 2014 dans l'état du Gujarat. Au cours des 10 premiers mois, ce service d'assistance téléphonique est venu en aide à 9 767 personnes, dont 8 654 disaient être des femmes. Sur la totalité des appels, 79 % (7 694) ont nécessité une intervention par téléphone ou en personne le jour de l'appel, et 43 % des appels (4 190) ont été réalisés par ou pour des femmes victimes de violences.

**Leçons tirées:**

Même si les données jusqu'alors disponibles montrent que les femmes victimes de violences sexistes ne cherchent que rarement de l'aide auprès d'interlocuteurs officiels, dans le Gujarat, les femmes ont réellement utilisé cette ligne téléphonique pour évoquer un grand nombre de problèmes, sur tout le spectre des violences faites aux femmes. Néanmoins, pour évaluer l'impact de cette ligne téléphonique, les définitions effectives des catégories de problèmes doivent être clarifiées. Le système de triage initial des appels entrants a été bénéfique pour traiter de gros volumes d'appels, mais il a peut-être contribué à la coupure de certaines communications.

## Resumen

**Situación:**

Es posible que muchas mujeres que sufren violencia de género nunca busquen ayuda formal, pues no se sienten seguras o no confían en que reciban ayuda si lo intentan.

**Enfoque:**

Una colaboración de los sectores público, privado y académico en Gujarat (India) estableció una línea de ayuda telefónica gratuita (denominada 181 Abhayam) para mujeres que sufrían violencia de género. La colaboración utilizó la infraestructura de servicio de respuesta de emergencia existente para conectar a las mujeres con asesoría telefónica, organizaciones no gubernamentales (ONG) y programas gubernamentales.

**Marco regional:**

En India, la prevalencia constante de violencia de género es del 37,2%, pero menos del 1% de las mujeres buscarán ayuda más allá de su familia o amigos. Antes de implementar la línea de ayuda, no existía este tipo de líneas gratuitas ni de sistemas de coordinación centralizados para programas gubernamentales, ONG y servicio de respuesta de emergencia.

**Cambios importantes:**

En febrero de 2014, se lanzó la línea de ayuda en todo Gujarat. Durante los 10 primeros meses, esta línea ayudó a 9 767 individuos, de los cuales 8 654 se identificaron como mujeres. De todas las llamadas, el 79% (7 694) requirieron una intervención telefónica o en persona ese mismo día, y el 43% (4 190) de las llamadas fueron realizadas por o para mujeres que sufrían violencia.

**Lecciones aprendidas:**

A pesar de que datos anteriores mostrasen que las mujeres que sufren violencia de género en muy raras ocasiones buscaban ayuda de fuentes formales, las mujeres de Gujarat sí que utilizaron la línea de ayuda en relación con temores sobre violencia de género. No obstante, para evaluar el impacto de la línea de ayuda, aún deben aclararse las definiciones operativas de las categorías de los temores. El primer sistema de clasificación de las llamadas entrantes resultó beneficioso para poder gestionar los elevados volúmenes de llamadas, pero es posible que haya contribuido a que se realicen menos llamadas.

## ملخص

**المشكلة:**

قد لا تسعى معظم النساء اللاتي يتعرضن لأشكال العنف القائمة على نوع الجنس إلى تلقي أية مساعدة رسمية أبدًا بسبب عدم شعورهن بالأمان أو الثقة بأنهن سيحصلن على المساعدة إذا ما سعين للحصول عليها.

**الأسلوب:**

قامت إحدى الشراكات الأكاديمية العامة الخاصة في ولاية غوجارات بالهند بتأسيس خط المساعدة الهاتفي المجاني – الذي يُطلق عليه اسم "‎‎181 Abhayam" – للنساء اللاتي يتعرضن لأشكال العنف القائم على نوع الجنس. واستعانت الشراكة بالبنية الأساسية القائمة بالفعل والمتمثلة في خدمة الاستجابة السريعة لحالات الطوارئ لربط النساء بمتخصصي الاستشارة الهاتفية والمنظمات غير الحكومية (NGO) وبرامج الحكومة.

**المواقع المحلية:**

تصل نسبة حالات العنف القائم على نوع الجنس إلى ‏37.2% في الهند، إلا أن نسبة النساء اللاتي يسعين إلى الحصول على المساعدة من أشخاص بخلاف أفراد عائلاتهن أو أصدقائهن لا تتعدى ‏1%. فقبل تقديم خط المساعدة، لم تكن هناك خطوط للمساعدة الهاتفية المجانية أو أنظمة مركزية تهدف لتنسيق برامج الحكومة والمنظمات غير الحكومية وخدمة الاستجابة السريعة لحالات الطوارئ.

**التغيّرات ذات الصلة:**

تم في شهر فبراير/شباط من عام 2014 طرح خط المساعدة الهاتفية عبر ولاية غوجارات. وفي العشرة شهور الأولى، قام خط المساعدة الهاتفية بمساندة 9767 فردًا، وقام 8654 شخصًا منهم بالتعريف عن أنفسهم بأنهم من النساء. ومن بين جميع المكالمات، حملت نسبة 79‏‏% (7694) منها طلبات للتدخل عن طريق الهاتف أو شخصيًا في نفس اليوم الذي تم فيه الاتصال، كما أن 43‏‏% (4190) من المكالمات وردت من سيدات أو لأجل سيدات يتعرضن إلى شكل من أشكال العنف.

**الدروس المستفادة:**

على الرغم من البيانات السابقة التي أظهرت أن النساء اللاتي يتعرضن لأشكال العنف القائم على نوع الجنس نادرًا ما يسعين إلى الحصول على المساعدة من مصادر رسمية، فإن النساء في ولاية غوجارات استخدمن بالفعل خط المساعدة لاهتمامهن بحالات العنف القائم على نوع الجنس. ومع ذلك، تحتاج التعاريف الإجرائية للفئات محل الاهتمام إلى مزيد من التوضيح، لتقييم الآثار المترتبة على خط المساعدة، فقد كان نظام التصنيف الأولي للمكالمات الواردة مفيدًا في التعامل مع عدد المكالمات المرتفع، إلا أنه من المحتمل أن يكون قد أسهم في نسبة المكالمات التي تعرضت إلى القطع قبل إتمامها.

## 摘要

**问题:**

许多遭遇性别暴力侵害的妇女可能永远都不会通过任何正式渠道寻求帮助，因为她们没有安全感，也没有信心在求助后能得到帮助。

**方法:**

印度古吉拉特邦的一项公私学术合作伙伴机制建立了一个免费求助热线——名为 181 Abhayam——专为正在遭受性别暴力侵害的妇女而设。 该合作伙伴机制利用现有的应急服务基础设施为妇女提供电话咨询、接触非政府机构 (NGO) 和政府项目的渠道。

**当地状况:**

在印度，遭受性别暴力侵害的终生发生率高达 37.2%， 但是只有 1% 以下的妇女向家人或朋友之外的人求助。 在该求助热线建立起来之前，当地并没有免费求助电话和政府项目、非政府组织以及应急服务的集中协调系统。

**相关变化:**

2014 年 2 月，求助热线在整个古吉拉特邦建立了起来。 在最初 10 个月中，该求助热线帮助了 9767 人，其中有 8654 人自称是妇女。 在所有来电中， 有 79% (7694) 的来电者请求在来电当天通过电话或派人亲自到现场进行干预；43% (4190) 的来电者是遭受性别暴力侵害的妇女或者为其求助的人。

**经验教训:**

尽管先前的数据显示遭受性别暴力侵害的妇女很少向正式渠道寻求帮助的情况，但是古吉拉特邦的妇女的确出于对不同程度性别暴力的担忧而充分利用了该求助热线。 然而，要评估该求助热线的影响力，则需要对不同类别担忧的操作性定义进行进一步的阐明。 来电的最初分类系统对接收大批量的来电颇为有利，但是可能会导致来电中断。****

## Резюме

**Проблема:**

Многие женщины, подвергающиеся гендерному насилию, никогда не обращаются за какой-либо организованной помощью, поскольку не чувствуют себя в безопасности или не уверены, что получат помощь, если попытаются обратиться за ней.

**Подход:**

В рамках партнерства между государственными, частными и академическими учреждениями в штате Гуджарат, Индия, была создана специальная линия под названием «181 Abhayam» (181 Абхаям) для предоставления информации и оказания помощи женщинам, подвергающимся гендерному насилию, с бесплатным телефонным номером. Партнерство использовало существующую инфраструктуру службы чрезвычайного реагирования в качестве промежуточного звена между женщинами и службами телефонного консультирования, неправительственными организациями (НПО) и государственными программами.

**Местные условия:**

В Индии гендерному насилию подвергаются 37,2% женщин, но менее 1% из них обращается за помощью к кому-либо вне своего близкого окружения (членов семьи или друзей). До внедрения специальной линии отсутствовали какие-либо подобные службы с бесплатным телефонным номером или централизованные системы координации для государственных программ, НПО и службы чрезвычайного реагирования.

**Осуществленные перемены:**

В феврале 2014 года была запущена специальная линия для предоставления информации и оказания помощи во всем штате Гуджарат. В первые 10 месяцев благодаря этой специальной линии была оказана помощь 9767 лицам, 8654 из которых были женщинами. 79% (7694) из числа всех звонящих потребовалось вмешательство по телефону или личная встреча в день звонка, в 43% (4190) случаев звонки были совершены женщинами, подвергающимися насилию, или от их имени.

**Выводы:**

Хотя полученные ранее данные свидетельствовали о том, что женщины, подвергающиеся гендерному насилию, редко обращаются за помощью в официальные органы, жительницы штата Гуджарат в действительности использовали специальную телефонную линию для решения разнообразных проблем, связанных с гендерным насилием. Тем не менее для оценки влияния специальной линии для предоставления информации и оказания помощи необходимо дополнительно уточнить рабочие определения категорий проблем. Первоначальная система фильтрации входящих звонков позволила успешно обрабатывать значительные объемы звонков, но также могла привести к увеличению числа сброшенных вызовов.

## Introduction

Globally, over one-third of all women will experience gender-based violence in their lifetime.^1^ Such violence includes any act that results in, or is likely to result in, physical, sexual or psychological harm or suffering to women, which includes coercion or arbitrary deprivation of liberty.^2^ Gender-based violence may be perpetrated by current or former partners, strangers, acquaintances or family members. Women who experience such violence may experience physical and mental health problems across their lifetime,^3^^,^^4^ including an increased risk of sexually transmitted infections,^5^ depression and anxiety,^6^ giving birth to low-birth-weight infants,^7^ and hypertension.^8^ While governments and nongovernmental organizations (NGOs) work to build support services and the legal infrastructure to prevent and to confront gender-based violence, women often do not seek out or cannot connect to these resources.

In India, there is a 37.2% lifetime prevalence of gender-based violence among women.^9^ Only 31.5% of these women will ever report gender-based violence to anyone, and less than 1% will report it to a formal source, such as the police or a physician.^10^ This is below the low global average of 7% of women reporting gender-based violence to any formal source.^10^

In January 2013, the Indian Department of Telecommunications released the toll-free phone number 181 across India, and mandated that every state government develop infrastructure for this number that would address the needs of women experiencing gender-based violence. This paper describes a multisectoral collaboration in the state of Gujarat, India, that has met this mandate by creating a toll-free helpline called 181 Abhayam.

## Approach

The Indian Administrative Service Secretary for Gujarat, Anju Sharma, who was also the state head of the Women and Child Development Department, brought together key collaborators across sectors to establish the helpline. For ensuring the effectiveness of the interventions and for creating accountability, the secretary enlisted the Government of Gujarat Home Department, which is responsible for the police. The secretary recruited the GVK Emergency Management Research Institute to be responsible for the implementation and operations of the helpline. The institute is the largest provider of free emergency medical services in India, covering 15 states. It operates as a public–private, not-for-profit partnership with each state’s government. The secretary also invited the Tata Institute of Social Sciences, which has a history of gender-based violence research, to provide technical expertise for helpline protocols.

The collaborators created a shared vision: any woman, irrespective of caste, religion, class, ability, age, sexual or political orientation, educational or economic status, would be able and empowered to seek and receive emergency services anywhere, at any time. Their mission – paid for by the Government of Gujarat – was to provide a coordinated, effective and timely multi-agency response that offered quality mental health, social, and legal services. They agreed upon a target group that reached beyond the traditional definition of gender-based violence: women in distress, which includes any woman who has or is experiencing psychological, emotional, financial, and/or social crisis or physical harassment. Here, crisis is defined as when a woman’s sense of self or safety is likely to be compromised.

To inform the direction of the helpline, the GVK Emergency Management Research Institute conducted a survey across three cities in which they intended to pilot services. Together, these cities had a total population of 4 661 804 women in 2011. The survey included 8935 participants (93%; 8269 women). Overall, 43% (3821) had experienced at least one type of harassment: 51% (4520) verbal harassment, 27% (2378) physical assault, 17% (1529) sexual assault and 27% (2438) stalking. The majority of respondents did not call the police when experiencing harassment (80%; 7146) and almost all respondents (95%; 8511) wanted an additional agency for gender-based violence-related support. Moreover, participants desired a range of services, such as counselling, police-assisted rescue and legal aid.

The helpline's central operations were based in the GVK Emergency Management Research Institute’s call centre. The collaborators recruited women with education and experience in social work to become response officers for the helpline. To prepare response officers to answer calls and respond to callers’ concerns, the collaborators jointly developed a month long training course. After successful training, a team of 32 female response officers were hired to keep the helpline staffed 24 hours a day, seven days a week.

The collaborators developed specific protocols for the response officer when answering calls. First, response officers triage calls. The protocols outline specific actions based on (i) the caller’s concern; (ii) the caller’s safety and the urgency of the concern; and (iii) the parameters of established governmental and NGO resources. Second, if the caller’s needs cannot be quickly met or if the call is emergent, then the call is transferred to a counsellor. Counsellors are the same aforementioned response officers, but acting in a specific role wherein they have more time to provide in-depth counselling and interventions to the woman calling. Response officers and counsellors, each independently classify every caller’s primary concern according to a predefined list of 55 concerns.

Response officers use the infrastructure of the call centre to connect women via phone directly to NGOs and governmental partners. Using the call centre technology, they are able to stay on the line with the caller to ensure that women obtain the help they need. Occasionally, response officers are unable to connect women to appropriate resources. If this occurs when a woman is in an emergent circumstance, then they may use escalation protocols. These protocols involve state level officers, within the home department and the women and child development department, who ensure that women receive the help they need.

In two of the cities and one district the helpline piloted rescue services. The rescue services consisted of a specifically designated van, driver, female police constable from the home department and a field response officer from the helpline. The team provided on-site counselling, mediation and rescue services. Via the helpline, callers could either directly ask for on-scene rescue services or counsellors could recommend the service to the caller.

All services, whether provided on the phone or in the field, are explained by response officers and counsellors and they require a caller’s verbal informed consent to proceed. A woman’s right to privacy is paramount throughout the process. Data from calls are stored on a central server, only accessible by helpline staff.

## Outcome

**Fig. 1:**
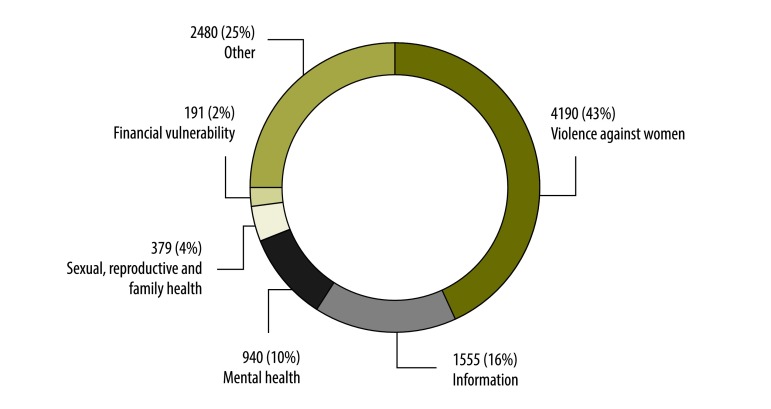
Distribution of callers’ concerns to a gender-based violence helpline in Gujarat, India, 2014

Note: Calls classified by response officers. For 32 calls, the response officer did not classify the caller’s concern. This data is considered missing.

The helpline was launched on 4 February 2014. A review of its first 10 months of operation demonstrated that the helpline reached women in distress. During this period, it received 9767 calls. Most callers identified themselves as women (8654). Many were married (5161) and 5479 callers called on their own behalf. The median age was 30 years (interquartile range: 24–38). Although the helpline targeted women, men made 364 calls on behalf of others and 304 calls on their own behalf; 301 did not identify who they were calling for. The caller’s primary concern, as classified by the response officer and the counsellor independent of each other, fell into one of six major categories: violence against women; financial vulnerability; mental health; sexual, reproductive and family health; information; and others (Fig. 1). Overall, 79% (7694) of calls required an intervention beyond information alone (Table 1).

**Table 1:** Types of calls to a gender-based violence helpline, Gujarat, India, 2014

Call type	Urgency	No. of calls (*n* = 9767), (%)	
		Response officer	Counsellor
Information	Low	1806 (18.5)	–
Linkage^a^	Low	267 (2.7)	–
Nonphysical assault	Low	3161 (32.4)	2768 (28.3)
Acute	Moderate	1365 (14.0)	1296 (13.3)
Severe	Moderate	2365 (24.2)	1861 (19.1)
Urgent	High	518 (5.3)	490 (5.0)
Emergent	High	285 (2.9)	525 (5.4)
Other	–	–	354 (3.6)
Missing^b^	–	–	2473 (25.3)

^a^ Linkage refers to calls where the concern is of low urgency and the help required is a linkage to a nongovernmental organization or government resource.

^b^ Missing records included 2133 calls of low urgency that may have been closed by the response officer. The remaining 340 calls were of moderate or high urgency and are considered dropped calls.

Note: A person who calls the helpline first talks to a response officer. If the caller’s needs cannot be quickly met or if the call is emergent, the response officer transfers the call to a counsellor.

Despite limited poster advertisements, the helpline received calls from all districts in Gujarat. The highest call volume (7245) came from the five most urban districts in Gujarat, while the lowest call volume (194) came from the six most rural districts. There could be several reasons for this, including a lack of marketing and less dense signage in more rural areas. Telephone coverage has not been a major obstacle in rural locations for GVK Emergency Management Research Institute’s emergency medical service operations, which uses the same infrastructure.

In March 2015, using the existing emergency response infrastructure, rescue services were expanded across Gujarat with 43 vans. An additional 110 response officers were hired to staff the expansion. In the subsequent nine months, helpline call volume increased, resulting in 73 238 helpline calls. Since the launch, the helpline has dispatched rescue services for over 18 000 of these calls.

## Lessons learnt

Previous data showed women who experienced gender-based violence rarely sought help from formal sources; however, women in Gujarat did use the helpline for concerns across the spectrum of gender-based violence. Multisectoral helplines like 181 Abhayam, which offer both counselling and connection to other partners, may serve as a central source of help and an intermediary for traditional formal sources of help (Box 1).

Box 1 **Summary of main lessons learnt**

- Despite previous data that showed women experiencing gender-based violence rarely sought help from formal sources, women in Gujarat did use the helpline for concerns across the spectrum of gender-based violence.
- When women call, their concerns are multifaceted and may require counselling for mental health support, help with legal redress and information about further support.
- To handle high call volumes, triaging was used to be able to quickly identify critical calls. However, the process increases the risk of dropped calls in the transition to the second tier.

Response officers and counsellors classified the callers’ concerns differently. For example, we found that if a call was classified by a response officer as violence against women, the counsellor’s reclassification predominately fell into information, mental health or other categories. This discrepancy, which repeated across categories, likely reflects the multifaceted nature of gender-based violence. Women call not only to report a particular act, but also to receive mental health counselling and to find legal redress. To better understand the full extent of women’s needs, we have changed the data collection process by creating clearer categories and allowing calls to be assigned multiple concern categories.

Even though triaging calls was effective, it may have created dropped calls by extending the length of the call and adding a call transfer. An estimated 727 calls were dropped in transfers between response officers and counsellors. Once staffing better matches call volume, the helpline plans to move away from the current triage system to decrease the number of dropped calls.

## Next steps

This paper demonstrates the feasibility of a multisectoral collaboration to leverage existing resources to connect women to available gender-based violence services. More work is needed to demonstrate the impact of 181 Abhayam on women’s ability to obtain services they are connected with, as well as the longer-term impact on their own health and behaviours. The collaborators are working to strengthen their follow-up process to be able to track outcomes, such as successful linkages to services and women’s experience with the helpline. This data will guide efforts to improve and expand services offered across the social service and public health sectors.

The key components of the 181 Abhayam model exist in at least the 15 Indian states where the GVK Emergency Management Research Institute operates, including the same emergency response infrastructure, state-level women and child development departments and home departments. The states also have the same national mandate to staff a 181 helpline. As in Gujarat, states will need a central leader who can bring this infrastructure and key stakeholders together to create a shared vision and to launch a multisectoral model that helps women who experience gender-based violence.
